# Multicentre prospective phase II trial of gefitinib for advanced non-small cell lung cancer with epidermal growth factor receptor mutations: results of the West Japan Thoracic Oncology Group trial (WJTOG0403)

**DOI:** 10.1038/sj.bjc.6604249

**Published:** 2008-02-19

**Authors:** K Tamura, I Okamoto, T Kashii, S Negoro, T Hirashima, S Kudoh, Y Ichinose, N Ebi, K Shibata, T Nishimura, N Katakami, T Sawa, E Shimizu, J Fukuoka, T Satoh, M Fukuoka

**Affiliations:** 1 Outpatients Treatment Center, National Cancer Center Hospital, 5-1-1, Tsukiji, Chuo-ku, Tokyo 104-0045, Japan; 2 Department of Medical Oncology, Kinki University School of Medicine, 377-2, Ohno-higashi, Sayama, Osaka 589-8511, Japan; 3 Department of Clinical Oncology, Osaka City General Hospital, 2-13-22, Miyakojima-hondori, Miyakojima, Osaka 534-0021, Japan; 4 Department of Thoracic Oncology, Hyogo Cancer Center, 13-70, Akashi, Kitaouji, Hyogo 673-8558, Japan; 5 Department of Thoracic Malignancy, Osaka Prefectural Medical Center for Respiratory and Allergic Diseases, 3-7-1, Habikino, Habikino, Osaka 583-8588, Japan; 6 Department of Respiratory Medicine, Osaka City University Medical School, 1-5-7, Asahi, Abeno, Osaka 545-8586, Japan; 7 Department of Thoracic Oncology, National Kyusyu Cancer Center, 3-1-1, Nodame, Minami, Fukuoka 811-1347, Japan; 8 Department of Respiratory Medicine, Iizuka Hospital, 3-83, Yoshio, Iizuka, Fukuoka 820-8505, Japan; 9 Department of Medicine, Koseiren Takaoka Hospital, 5-10, Eiraku, Takaoka, Toyama 933-8555, Japan; 10 Division of Respiratory Medicine, Kobe City General Hospital, 4-6, Minatojima-nakamachi, Chuo-ku, Kobe, Hyogo 650-0046, Japan; 11 Department of Integrated Oncology, Institute of Biomedical Research and Innovation, 2-2, Minatojima-minamimachi, Chuo-ku, Kobe, Hyogo 650-0047, Japan; 12 Department of Respiratory Medicine, Gifu Municipal Hospital, 7-1, Kashima, Gifu 500-8323, Japan; 13 Division of Medical Oncology and Respiratory Medicine, Faculty of Medicine, Tottori University, 36-1, Nishi-machi, Yonago, Tottori 683-8504, Japan; 14 Laboratory of Pathology, Toyama University Hospital, Toyama, 2630, Sugitani, Toyama 930-0194, Japan; 15 Department of Medical Oncology, Kinki University School of Medicine, Sakai Hospital, 2-7-1, Harayamadai, Minami-ku, Sakai, Osaka 590-0132, Japan

**Keywords:** epidermal growth factor receptor (*EGFR*) mutation, gefitinib, non-small cell lung cancer (NSCLC), multicentre prospective phase II, central laboratory

## Abstract

The purpose of this study was to evaluate the efficacy of gefitinib and the feasibility of screening for epidermal growth factor receptor (*EGFR*) mutations among select patients with advanced non-small cell lung cancer (NSCLC). Stage IIIB/IV NSCLC, chemotherapy-naive patients or patients with recurrences after up to two prior chemotherapy regimens were eligible. Direct sequencing using DNA from tumour specimens was performed by a central laboratory to detect *EGFR* mutations. Patients harbouring *EGFR* mutations received gefitinib. The primary study objective was response; the secondary objectives were toxicity, overall survival (OS), progression-free survival (PFS), 1-year survival (1Y-S) and the disease control rate (DCR). Between March 2005 and January 2006, 118 patients were recruited from 15 institutions and were screened for *EGFR* mutations, which were detected in 32 patients – 28 of whom were enrolled in the present study. The overall response rate was 75%, the DCR was 96% and the median PFS was 11.5 months. The median OS has not yet been reached, and the 1Y-S was 79%. Thus, gefitinib chemotherapy in patients with advanced NSCLC harbouring *EGFR* mutations was highly effective. This trial documents the feasibility of performing a multicentre phase II study using a central typing laboratory, demonstrating the benefit to patients of selecting gefitinib treatment based on their *EGFR* mutation status.

Gefitinib, a tyrosine kinase inhibitor (TKI), is an orally active small molecule that functions as a selective epidermal growth factor receptor (*EGFR*) inhibitor (Ranson *et al*, 2002). Two phase II trials (Fukuoka *et al*, 2003; Kris *et al*, 2003) for previously treated non-small cell lung cancer (NSCLC) (IDEAL-1 and -2, respectively) have documented favourable objective responses in 14–18% of patients. However, in a phase III trial (Thatcher *et al*, 2005), no survival benefit of gefitinib was observed when compared with best-supportive care (BSC) for previously treated NSCLC. In contrast, we have seen a significant survival benefit of erlotinib compared with BSC as a salvage therapy (BR21); erlotinib is also an *EGFR*-TKI and its chemical structure, which is based on quinazoline, is quite similar to that of gefitinib (Shepherd *et al*, 2005). Although we do not know whether differences between gefitinib and erlotinib were responsible for these different outcomes, appropriate patient selection to identify good responders is likely crucial for revealing the clinical benefits of the *EGFR*-TKI family.

Patient subset analyses of these randomised phase III trials or retrospective trials (Kaneda *et al*, 2004; Miller *et al*, 2004) clearly show the existence of populations that are more likely to respond to gefitinib and erlotinib, including women, patients with adenocarcinoma (especially with bronchial alveolar carcinoma (BAC)), nonsmokers and Asian patients (compared with Caucasians). Somatic mutations in specific regions of exons 18, 19 and 21 of the ATP-binding domain of *EGFR* have recently been shown to have strong associations with sensitivity to gefitinib or erlotinib (Lynch *et al*, 2004; Paez *et al*, 2004; Pao *et al*, 2004). Consistent with these findings, the frequencies of these *EGFR* mutations were higher in women, patients with adenocarcinoma, nonsmokers and Asians, all of whom are among the more frequent responders, as mentioned above (Shigematsu *et al*, 2005). There are two characteristic types of *EGFR* mutations. One is the presence of in-frame deletions, including the amino acids at codons 746–750 in exon 19, and the other is an amino-acid substitution at codon 858 (L858R) in exon 21. Recent analyses (Bell *et al*, 2005) of phase II and III trials for *EGFR-*TKI, in which patients were not selected based on their mutation status, have suggested that *EGFR* mutations are correlated with response to therapy but are not correlated with overall survival (OS). Furthermore, *EGFR* gene amplification/copy number (Cappuzzo *et al*, 2005; Hirsch *et al*, 2005) or overexpression (Hirsch *et al*, 2003) has been shown to be a more useful prognostic marker of response to gefitinib treatment. Patient selection according to *EGFR* mutation status may yield a superior survival rate by excluding patients who are unlikely to respond to gefitinib treatment. However, other populations that might obtain a clinical benefit from gefitinib treatment, even in the absence of *EGFR* mutation, may exist.

Three Japanese groups (Asahina *et al*, 2006; Inoue *et al*, 2006; Yoshida *et al*, 2007) have reported prospective phase II studies of gefitinib for advanced-stage NSCLC that were designed to consider the *EGFR* mutation status of the patients. All of these studies have reported a high response rate and extended progression-free survival (PFS) period, compared with historical controls. However, all of these studies had a relatively short observation period, making the data preliminary. Moreover, the original sample size was calculated after patient selection, and a critical consideration of the suitability of the assay used to detect the mutations (which was performed using small paraffin-embedded specimens obtained from bronchoscopic biopsies), and the estimated *EGFR*-positive rate were lacking. Additionally, all the trials were conducted at single institutions located in one small area of Japan. Thus, the published data may not be representative of the situation found in general clinical practice throughout Japan and therefore may not directly translate to the general feasibility of gefitinib treatment in Japan.

In view of this situation, we performed a multicentre prospective phase II trial of gefitinib for advanced NSCLC harbouring *EGFR* mutations. We prospectively registered patients from 15 different institutes in Japan at the beginning of *EGFR* mutation screening using a central database. Whether or not tissue was available from a bronchoscopic biopsy or surgery was not an inclusion criterion. All the clinical samples from the registered patients were delivered to a central laboratory that then determined the *EGFR* mutation status or the histological BAC features. The analysis of the survival data was based on a minimum observation period of at least 15 months from the time of entry of the last patient.

## MATERIALS AND METHODS

### Eligibility criteria

Eligible patients had histologically confirmed stage III NSCLC for which thoracic irradiation was not indicated or were stage IV. Chemotherapy-naive patients or those who had previously received up to two prior chemotherapy regimens, including those performed in an adjuvant setting, were eligible. Other eligibility criteria included an age ⩾20 years, measurable disease, the availability of sufficient amounts of tumour specimen for *EGFR* mutation analysis, an Eastern Cooperative Oncology Group performance status of 0–2, adequate organ function (WBC⩽3000 *μ*l^−1^, platelets⩾75 000 *μ*l^−1^, AST and ALT⩽100 IU l^−1^, serum creatinine⩽twice the upper limit of the reference range; *P*_aO2_⩾60 mm Hg). The exclusion criteria included pulmonary fibrosis, the presence of symptomatic brain metastasis, active concomitant malignancy, severe heart disease, active gastrointestinal bleeding and continuous diarrhoea. All the patients signed a written informed consent form. Approval of this study and the gene analyses were obtained from the Institutional Review Board and the Ethics Committee of each hospital.

### *EGFR* gene analysis

Tumour specimens were obtained using bronchial fiberscope or surgical procedures. The specimens were fixed with formalin and embedded in paraffin. Four slices (4–5 *μ*m) from the embedded block were sent to a central laboratory (Mitsubishi Chemical Safety Institute Ltd., Ibaraki, Japan) for genetic analysis. Most of the tumour specimens were available prior to the registration of this study. Genomic DNA was isolated from specimens using QIAamp Micro kits (QIAGEN KK, Tokyo, Japan). The *EGFR* mutations in exons 18, 19 and 21, as previously reported (Lynch *et al*, 2004; Paez *et al*, 2004), were determined using polymerase chain reaction (PCR) amplification and intron–exon boundary primers according to the published method. An *EGFR* registrant mutation in exon 20, which was reported by Pao *et al* (2005) was also examined using PCR and the previously reported primers. Polymerase chain reaction was performed using a Gene Amp PCR System 9700 (Applied Biosystems, Foster City, CA, USA), and the PCR products were confirmed using a Bioanalyzer 2100 (Agilent Technologies Inc., Santa Clara, CA, USA), then sequenced directly using the Big Dye Terminator v3.1 Cycle Sequencing Kit (Applied Biosystems) and ABI PRISM 3100 (Applied Biosystems). All sequencing reactions were performed in both forward and reverse directions and were analysed using the Basic Local Alignment Search Tool (BLAST); all the electropherograms were reanalysed by visual inspection to check for mutations. The presence of an *EGFR* mutation was confirmed using at least three independent PCR.

All sequence data were sent from the central laboratory to Kinki University. A principle investigator then confirmed whether or not the *EGFR* mutation status was positive, and the results were sent to the West Japan Thoracic Oncology Group (WJTOG) data centre. The data centre then informed each participating centre of the results of the genetic analysis and requested that the eligibility criteria of the patients be rechecked to insure that only *EGFR-*positive subjects were registered in the trial. Each tumour was categorised according to histology by a pulmonary pathologist (JF). The percentage of area exhibiting a BAC pattern was also examined to determine the WHO pathological category.

### Treatment plan

Gefitinib (250 mg day^−1^) was administered once daily. Treatment was continued uninterrupted until disease progression or intolerable toxicity (grade 4 nonhaematological toxicities, any incidents of interstitial pneumonia or a treatment delay of more than 2 weeks because of adverse effects). Gefitinib administration was delayed if the patient's leukocyte and platelet counts were lower than 1500 and 5000 *μ*l^−1^, respectively, and was withheld until these counts had recovered. Gefitinib administration was also delayed if grade 3 or greater nonhaematological toxicities without nausea, vomiting or alopecia occurred and was withheld until recovery to grade 2.

Routine clinical and laboratory assessments and chest X-ray assessments were performed weekly or biweekly, where possible; CT examinations of the target lesion were performed every month, and magnetic resonance imaging of the whole brain and a bone scan were performed every 3 months. The objective responses of the patients were evaluated every month using the Response Evaluation Criteria in Solid Tumours (RECIST) guidelines (Therasse *et al*, 2000). Tumour response was centrally evaluated by independent reviewers at an extramural conference and was performed for the intent-to-treat population. All adverse effects that occurred during gefitinib treatment were reported, and the severity of the effects was graded according to the National Cancer Institute Common Terminology Criteria for Adverse Events, version 3.0.

### Statistical analyses

The primary end point of this study was the response rate. A one-stage design using the binominal probability was used to determine the sample size. Assuming that a response rate of 50% would indicate potential usefulness, whereas a rate of 25% would be the lower limit of interest, and with *α*=0.10 (two side) and *β*=0.20, the estimated accrual number was 23 patients. Estimating that the *EGFR-*positive rate would be about 20%, the screening number required to accrue 23 *EGFR-*positive patients was 115. After assuming an inevaluability rate of <10%, the final required screening number was 125.

The secondary end points of this study were toxicity, OS, PFS, 1-year survival (1Y-S) and the disease control rate (DCR). Survival analyses were conducted on the intent-to-treat population using follow-up data available as of 30 April 2007. The survival curves were estimated using Kaplan–Meier plots.

## RESULTS

### Patient characteristics

Between March 2005 and January 2006, 118 patients were prospectively screened from 15 institutions; 117 of them underwent *EGFR* mutation analysis (tumour tissue was not available for one patient). The median time required for the *EGFR* mutation analysis was 12 days (range: 7–28 days). Among the 117 patients, *EGFR* mutations were detected in 32 patients (27%), 14 of whom had a deletion in or near E746-A750 (including one del E746-T751 ins A, two del L747-T751 and one del L747-T753 ins S) in exon 19. A further 17 had L858R, and one had a L861Q point mutation in exon 21 (Table 1).

Tissue samples from 17 patients (53%) were obtained by transbronchial biopsy. The *EGFR* detection rates for the surgical specimens and the bronchoscopic biopsy specimens were similar (30 *vs* 25%). The *EGFR* mutations were significantly more frequent in women (*P*⩽0.02), in patients with adenocarcinoma (*P*=0.001) and in people who had never smoked (*P*<0.001) (Table 2). Finally, 28 patients (14 with deletions in exons 19 and 14 with point mutations in exon 21) were actually registered and received treatment with gefitinib, whereas four patients were dropped from the study as they became ineligible because of tumour progression during the time required for the mutation analysis.

Patient characteristics are listed in Table 3. In the initial screening, there were 56 female patients (48%), 97 patients (83%) with adenocarcinoma and 53 (45%) who had never smoked. The frequency of these characteristics was higher among the patients with *EGFR* mutations who were actually registered; namely, 18 patients (64%) were women, 27 (96%) had adenocarcinoma and 19 (68%) had never smoked. The median age of the 28 actually registered patients was 68 years; 24 patients (86%) had a good performance status (0–1), 22 (79%) had stage IV diseases and 17 (61%) were chemotherapy naive. Thoracic irradiation was contraindicated in one patient with stage IIIA disease because of the large irradiation field that would have been required. All five patients with stage IIIB diseases had malignant effusions. Four patients had received adjuvant therapies; five had received platinum doublets or a combination of gemcitabine and vinorelbine as their first-line therapy. Two patients had received two regimens of platinum doublets followed by docetaxel or pemetrexed. One patient had received local radiation for pain control.

### Response and survival

The objective tumour responses are listed in Table 4. The overall response rate and DCR were 75% (95% CI: 57.6–91.0%) and 96% (95% CI: 87.0–96.4%), respectively. Five out of ten male patients (50%), six out of nine smokers (67%) and five out of eight male smokers with adenocarcinoma (63%) achieved a PR. One female nonsmoker with squamous cell carcinoma also achieved a PR. Among the registered patients with *EGFR* mutations, the response rate was no different between current/former smokers and those who had never smoked (67 *vs* 79%) or between chemotherapy-naive and postchemotherapy patients (77 *vs* 73%). Female and patients with a mutational deletion in exon 19 tended to have a higher response rate than male (89 *vs* 50%) and patients with a missense mutation in exon 21 (86 *vs* 64%), respectively.

**Figure 1:**
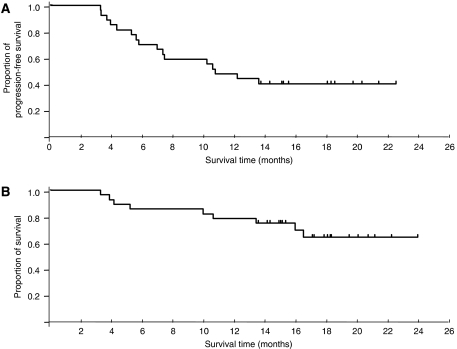
(**A**) Progression-free survival (PFS) and (**B**) overall survival (OS) of all eligible patients (*n*=28). The median PFS was 11.5 months. The median OS has not yet been reached. The 1-year survival rate was 79%.

The median follow-up time was 18.6 months (range: 13.8–23.4 months). The median PFS time was 11.5 months (95% CI: 7.3 months to -) (Figure 1A). The median OS has not yet been reached, and the 1Y-S was 79% (95% CI: 63.4–93.8%) (Figure 1B).

### Safety and toxicity

Toxicity was evaluated in all eligible patients (Table 5). The most frequent adverse events were rash, dry skin, diarrhoea, stomatitis and elevated AST/ALT levels. Two patients experienced grade 3 rash and one patient experienced grade 3 keratitis; however, these patients all achieved a PR, and the adverse effects subsided after pausing gefitinib treatment for around 2 weeks. Four patients experienced grade 3 hepatotoxicity; three of these patients had to discontinue treatment for this reason.

One patient developed interstitial lung disease (ILD) (Ando *et al*, 2006). Ground-glass opacity was detected in the right upper lobe 19 days after the start of gefitinib administration, resulting in the cessation of treatment. However, the lesion enlarged into bilateral lung fields on day 25, and steroid therapy was initiated. Nonetheless, the patient died of respiratory failure on day 48. Two patients also experienced grade 1 ILD. They recovered without steroid administration.

### Subsequent treatment after disease progression

Of the 14 patients who become refractory to gefitinib and exhibited disease progression, 10 received chemotherapy as their first treatment regimen after gefitinib (Table 6); 5 patients received platinum doublets and 1 patient received vinorelbine as a second-line treatment; and 3 received docetaxel and 1 received platinum doublet as a third-line treatment. In all, 4 out of the 10 patients (40%) had a PR. Of the nine patients who become refractory to the first treatment regimen after gefitinib, six received chemotherapy as their second regimen after gefitinib, including one who received gemcitabine, one who received docetaxel, and one who was re-treated with gefitinib as a third-line therapy; two other patients received docetaxel and one was re-treated with gefitinib as a fourth-line therapy. Two of the six patients (33%) had a PR. The two patients who received gefitinib re-treatment both had SD.

### BAC features, *EGFR* amplification and T790M mutation in exon 20

A total of 110 tissue samples were available for pathological review, of which 90 were from adenocarcinoma; 33 of these specimens (37%) revealed proportional BAC components in the specimen. Among them, 15 were considered extensive and the remaining 18 were found to have minor BAC components. The 39 surgical specimens included 36 from adenocarcinomas. The *EGFR* mutations were detected in 12 out of the 36 adenocarcinoma specimens. None of the samples with a BAC component, micropapillary pattern or mucin production was associated with an *EGFR* mutation (Table 7).

Data on *EGFR* gene copy numbers were available in only 12 samples. We used the criteria for defining a high *EGFR* gene copy number (gene amplification or high polysomy, as determined using FISH) that were described in a previous report (Cappuzzo *et al*, 2005). A total of 7 out of the 12 samples had a high gene copy number (FISH positive), and 6 (3 with *EGFR* mutations) out of the 7 samples had proportional BAC components. In all, 5 out of the 12 samples were FISH negative, only 1 (with no *EGFR* mutation) of which had a BAC component. Two patients that were FISH negative, BAC negative and *EGFR* mutation positive had SD when treated with gefitinib.

Another *EGFR* mutation, T790M in exon 20, has been reported to be associated with resistance to gefitinib (Kobayashi *et al*, 2005; Pao *et al*, 2005). We checked for this mutation in six patients who did not respond to gefitinib; however, the mutation could not be identified in any of the patients.

## DISCUSSION

We performed a multicentre phase II study examining the use of gefitinib for advanced NSCLC in patients with *EGFR* mutations, prospectively recruiting patients at the time of genetic screening and avoiding a selection bias. All patients were registered in a central database. All tissues were delivered from the local participants to the central facility, where they were reviewed by a pathology specialist and the *EGFR* mutation status was evaluated. The median time for the *EGFR* mutation detection analysis was 12 days, which is probably an acceptable time lag before the start of treatment for advanced NSCLC. However, a shorter period would clearly be desirable for routine clinical practice. Indeed, 4 out of the 32 *EGFR*-positive patients were dropped from the study because of disease progression before their actual registration could occur. Yatabe *et al* (2006) has developed a rapid assay to detect *EGFR* mutations, and we have decided to use this assay in a phase III trial. The *EGFR* mutation rates in transbronchial biopsy samples were found to be the same as those in surgical specimens, suggesting that this assay can also accommodate stage IV NSCLC. We detected the two characteristic types of *EGFR* mutations (in exons 19 and 21) in 44 and 56% of the patients, respectively (Table 1); these percentages are identical to those in previous reports from Japan (Shigematsu *et al*, 2005; Asahina *et al*, 2006; Inoue *et al*, 2006; Yatabe *et al*, 2006; Yoshida *et al*, 2007). In summary, we confirmed the feasibility of using the *EGFR* detection assay in daily practice.

The overall response rate was 75%, which was comparable to those of other phase II studies of gefitinib in patients with *EGFR* mutations (Asahina *et al*, 2006; Inoue *et al*, 2006), despite our study permitting the entry of patients who had previously received up to two chemotherapy regimens. The DCR of 96% was relatively high, and the median PFS of 11.5 months and 1Y-S of 79% were also very promising. In a Korean study, Lee *et al* (2006) also reported a very promising response rate (56%) and 1Y-S (76%) for gefitinib in a prospective study of selected NSCLC patients with adenocarcinoma and never/light smokers, defined as having smoked no more than 100 cigarettes during one's lifetime. In the screening process for the present study, *EGFR* mutations were significantly more frequent in women, patients with adenocarcinoma and those who had never smoked. However, among the patients who were selected according to their *EGFR* mutation status, no differences in response were observed between never smokers and current/former smokers or between chemotherapy-naive and postchemotherapy patients. In a retrospective study, Han *et al* (2006) directly compared clinical predictors (smoking history, gender and histology) and the *EGFR* mutation status for their ability to predict response and survival. They showed that female never smokers with adenocarcinoma (three clinical predictors) had a 33% response rate, whereas patients with a positive *EGFR* mutation status had a 62% response rate. Furthermore, in a multivariate analysis, only a positive *EGFR* mutation status was associated with an improved OS, suggesting that the *EGFR* mutation status should be analysed whenever possible to optimise response predictions based on clinical background factors. In the present study, *EGFR* mutations were detected in 16 out of 40 (40%) female never smokers with adenocarcinoma who underwent the screening process, and 14 out of these 16 patients (88%) achieved a response after undergoing gefitinib therapy. We could not compare the predictive powers of clinical predictors and the *EGFR* mutation status with regard to the clinical benefits of gefitinib in this study. Thus, the need for *EGFR* mutation testing among clinically favourable patients remains uncertain. Decisions regarding the first-line therapy of choice for patients with *EGFR* mutations or a clinically favourable profile (nonsmoker with adenocarcinoma) must also await the results of an ongoing randomised phase III study in an Asian population (IPASS: Iressa Pan-Asian Study) comparing platinum doublets with gefitinib.

In contrast, 50% of the men, 67% of the smokers and 63% of the men who were smokers achieved a PR in this study. Furthermore, one female nonsmoker with squamous cell carcinoma also responded to gefitinib. The histological type of this tumour was reassigned by a pulmonary pathologist, and the tumour was finally confirmed to be a squamous cell carcinoma. Squamous cell carcinoma harbouring an *EGFR* mutation is rarely seen but has been previously reported (Asahina *et al*, 2006). In a Japanese phase II trial of gefitinib for unselected chemotherapy-naive patients (Niho *et al*, 2006), the response rates among smokers, men, and patients with nonadenocarcinoma were 19, 13 and 10%, respectively. Thus, NSCLC patients who are either smokers, men or have a nonadenocarcinoma histology are unlikely to receive gefitinib treatment as a first-line treatment instead of standard chemotherapies (platinum doublets), which yield a response rate of about 30% (Schiller *et al*, 2002). Therefore, *EGFR* mutation screening may have a higher impact on the selection of responders to gefitinib treatment among these kinds of Asian patient subset (for example, smokers with adenocarcinoma, and nonsmoking men or women with nonadenocarcinoma).

The benefit of chemotherapy in general among patients with *EGFR* mutations, compared with *EGFR* mutation-negative patients, remains uncertain. Previous studies (Bell *et al*, 2005) have suggested that patients with *EGFR* mutations tend to be more sensitive to chemotherapy than those with wild-type *EGFR*. In the present study, 40 and 33% of the patients responded to first- and second-line chemotherapy regimens after gefitinib, respectively. These relatively high response rates for refractory NSCLC suggest that patients with an *EGFR* mutation-positive status are generally sensitive to chemotherapy. Large-scale multivariate analyses, using pooled data from prospective phase II or III trials in which the *EGFR* mutation status was clearly confirmed, are needed to clarify this point.

The toxicities observed in the present study were mostly tolerable. Most of the common adverse events, like rash, diarrhoea or hepatotoxicity, were mild and subsided after gefitinib administration was paused for a short period. One male smoker with adenocarcinoma died of ILD. Thus, even among patients who are selected based on their *EGFR* mutation status, men or smokers may still be at risk for developing ILD; therefore, biomarkers to predict ILD are needed.

Patients with exon 19 mutations tended to have a higher response rate than those with a missense mutation in exon 21, consistent with the findings of previous reports (Jackman *et al*, 2006; Riely *et al*, 2006). The Spanish Lung Cancer Group also reported on a prospective phase II study of erlotinib in advanced NSCLC patients with *EGFR* mutations (Paz-Ares *et al*, 2006). The overall response rate was 82%. They also showed a difference in response rates between patients with mutations in exons 19 and 21 (95 and 67%, respectively). Exon 11 c-kit mutations are more closely correlated with a good prognosis in patients with gastrointestinal stromal tumour, who may benefit from lower doses of imatinib, whereas patients with exon 9 mutations may require higher doses (Debiec-Rychter *et al*, 2006). In the case of *EGFR*, functional differences between mutation types may also exist.

We found no discernible associations between the *EGFR* mutation frequency and the presence of a BAC component. Several reports, including that of Hirsch *et al* (2005) suggest that a higher *EGFR* copy number is correlated with BAC histological features. We also found an association between a high *EGFR* copy number and the presence of a BAC component, even though the number of specimens examined was relatively small. In a study on erlotinib, the presence of a BAC component was clearly associated with *EGFR* amplification. As the *EGFR* mutation rate is lower in western populations than in Asian populations, the *EGFR* gene copy number might be a more useful biomarker in western populations, especially with regard to the use of erlotinib.

In conclusion, gefitinib treatment for patients with advanced NSCLC harbouring an *EGFR* mutation demonstrated a promising activity in patients with a good performance status. Patient screening according to *EGFR* mutation status may be a useful tool in daily practice and will likely have a great impact on the selection of patients who are likely to benefit from gefitinib treatment.

## Figures and Tables

**Table 1:** Type of *EGFR* mutations (*n*=32)

**Characteristics**	**No. of patients**	**%**
Exon 18	0	0
		
Exon 19	14	44
del E746-A750	10	32
del E746-T751 ins A	1	3
del L747-T751	2	6
del L747-T753 ins S	1	3
		
Exon 21	18	56
L858R	17	53
L861Q	1	3

EGFR=epidermal growth factor receptor.

**Table 2:** Relationship between patient characteristics and *EGFR* mutation status

	***EGFR* mutation positive (*n*=32)**		***EGFR* mutation negative (*n*=85)**		
**Characteristics**	**No. of Patients**	**%**	**No. of Patients**	**%**	***P***
*Sex*					
Male	11	34	50	59	
Female	21	66	35	41	<0.02
					
*Histology*					
Adenocarcinoma	31	97	66	78	
Nonadenocarcinoma	1	3	19	22	=0.001
					
*Smoking status*					
Never	21	66	31	36	
Current/former	11	34	54	64	<0.001

EGFR=epidermal growth factor receptor.

**Table 3:** Patient characteristics of all registered patients (*n*=28)

**Characteristics**	**No. of patients (%)**
*Age*	
Median	68
Range	49–89
	
*Performance status*	
0	11 (39)
1	13 (47)
2	4 (14)
	
*Sex*	
Male	10 (36)
Female	18 (64)
	
*Histology*	
Adenocarcinoma	27 (96)
Squamous cell carcinoma	1 (4)
Large cell carcinoma	0 (0)
Adenosquamous carcinoma	0 (0)
Other	0 (0)
	
*Smoking status*	
Never	19 (68)
Current/former	9 (32)
	
*Stage*	
IIIA^a^	1 (3)
IIIB	5 (18)
IV	22 (79)
	
*Prior cancer therapy*	
Chemotherapy	
No	17 (61)
One regimen (adjuvant)	4 (14)
One regimen (not adjuvant)	5 (18)
Two regimens	2 (7)
Recurrence after surgery	11 (39)
Radiation	1 (4)

a Unresectable, no indication for thoracic radiation because of a large radiation field.

**Table 4:** Response rate (*n***=**28)

**Response**	**No. of patients**	**Response rate (%)**	**95% CI**
Complete response	1	3.6	
Partial response	20	71.4	
Stable disease	6	21.4	
Progressive disease	0	0.0	
Not evaluable^a^	1	3.6	
Overall response	21	75.0	57.6–91.0
Disease control rate	27	96.4	87.0–96.4

CI=confidence interval.

a One patient was not evaluable because of a poor evaluation of efficacy.

**Table 5:** Common adverse events (*n***=**28)

	**No. of patients (%)**			
**Adverse events**	**Grade 1**	**Grade 2**	**Grade 3**	**Grade 4**
*Haematologic*				
Anaemia	12 (43)	3 (11)	0 (0)	0 (0)
Leucopaenia	4 (14)	1 (4)	2 (7)	0 (0)
Neutropaenia	4 (14)	1 (4)	1 (4)	0 (0)
Thrombocytopaenia	3 (11)	0 (0)	0 (0)	0 (0)
				
*Nonhaematologic*				
Rash	10 (36)	11 (39)	2 (7)	0 (0)
Dry skin	9 (32)	10 (36)	0 (0)	0 (0)
Nail changes	5 (18)	2 (7)	0 (0)	0 (0)
Keratitis	0 (0)	0 (0)	1 (4)	0 (0)
Fever	0 (0)	1 (4)	0 (0)	0 (0)
Fatigue	3 (10)	3 (10)	3 (10)	0 (0)
Diarrhoea	7 (25)	1 (4)	0 (0)	0 (0)
Constipation	1 (4)	0 (0)	0 (0)	0 (0)
Stomatitis	8 (29)	1 (4)	0 (0)	0 (0)
Gastritis	1 (4)	0 (0)	0 (0)	0 (0)
Anorexia	2 (7)	1 (4)	0 (0)	0 (0)
Nausea	3 (11)	1 (4)	0 (0)	0 (0)
Vomiting	2 (7)	2 (7)	1 (4)	0 (0)
Dyspnoea	2 (7)	0 (0)	1 (4)	0 (0)
ILD	2 (7)	0 (0)	0 (0)	1 (4)^a^
Vertigo	1 (4)	1 (4)	0 (0)	0 (0)
Dysgeusia	0 (1)	1 (4)	0 (0)	0 (0)
Elevated AST/ALT	10 (36)	2 (7)	4 (14)	1 (4)^a^
Elevated creatinine	2 (7)	1 (4)	2 (7)	0 (0)

ALT=alanine transaminase; AST=aspartate transaminase; ILD=interstitial lung disease.

a Same patient.

**Table 6:** Subsequent treatments after failure to respond to gefitinib (*n***=**28)

**Gefitinib treatment**	**No. of Patients**	**1st regimen after gefitinib**	**No. of patients**	**2nd regimen after gefitinib**	**No. of patients**
1st line	17	Plt doublet	5	Gem or Doce	2
				Gefitinib^a^	1
		VNR	1	—	-
2nd line^b^	4	Doce	2	Doce	1
		Plt doublet	1	Doce	1
2nd line	5	Doce	1	Gefitinib^a^	1
3rd line	2	—	—	—	—
Total	28		10		
Response			4/10		2/6

Doce**=**docetaxel; Gem**=**gemcitabine; Plt**=**platinum; VNR**=**vinorelbine.

a Both patients had an SD response after gefitinib re-treatment.

b First regimen as systemic chemotherapy after adjuvant treatment.

**Table 7:** Bronchial alveolar carcinoma (BAC) features and *EGFR* mutation status

	***EGFR* mutation**		
	+	−	***P-*value**
Surgically resected adenocarcinoma case	12	24	
			
*BAC component*			
Yes	8	17	1.0
No	4	7	
			
*Micropapillary pattern*			
Yes	4	12	0.48
No	8	12	
			
*Mucin production*			
Yes	1	5	1.0
No	11	19	

EGFR=epidermal growth factor receptor.
